# Spontaneous spheroids from alveolar bone-derived mesenchymal stromal cells maintain pluripotency of stem cells by regulating hypoxia-inducible factors

**DOI:** 10.1186/s40659-023-00421-w

**Published:** 2023-04-05

**Authors:** Ni Li, Xiaofeng Dai, Fei Yang, Yang Sun, Xingwen Wu, Qianrong Zhou, Kai Chen, Jian Sun, Wei Bi, Le Shi, Youcheng Yu

**Affiliations:** 1 Shanghai University of Medicine and Health Sciences Affiliated Zhoupu Hospital, Shanghai, China 201318,; 2 The College of Medical Technology, Shanghai University of Medicine and Health Sciences, Shanghai, China 201318; 3 Department of Stomatology, Zhongshan Hospital, Fudan University, Shanghai, China 180 Fenglin Road, 200032,; 4 Department of Stomatology, Shanghai Jing’an District Dental Clinic, Shanghai, China 15 Pingxingguan Road 200040,; 5 Department of Stomatology, Shanghai Tenth People’s Hospital, Tongji University School of Medicine, Shanghai, China 200072

**Keywords:** Alveolar bone, Spontaneous spheroid, Somatic stem cells, Pluripotency, Hypoxia-inducible factors

## Abstract

**Background:**

Spontaneous spheroid culture is a novel three-dimensional (3D) culture strategy for the rapid and efficient selection of progenitor cells. The objectives of this study are to investigate the pluripotency and differentiation capability of spontaneous spheroids from alveolar bone-derived mesenchymal stromal cells (AB-MSCs); compare the advantages of spontaneous spheroids to those of mechanical spheroids; and explore the mechanisms of stemness enhancement during spheroid formation from two-dimensional (2D) cultured cells.

**Methods:**

AB-MSCs were isolated from the alveolar bones of C57BL/6 J mice. Spontaneous spheroids formed in low-adherence specific culture plates. The stemness, proliferation, and multi-differentiation capacities of spheroids and monolayer cultures were investigated by reverse transcription quantitative polymerase chain reaction (RT-qPCR), immunofluorescence, alkaline phosphatase (ALP) activity, and oil-red O staining. The pluripotency difference between the spontaneous and mechanical spheroids was analyzed using RT-qPCR. Hypoxia-inducible factor (HIFs) inhibition experiments were performed to explore the mechanisms of stemness maintenance in AB-MSC spheroids.

**Results:**

AB-MSCs successfully formed spontaneous spheroids after 24 h. AB-MSC spheroids were positive for MSC markers and pluripotency markers (Oct4, KLF4, Sox2, and cMyc). Spheroids showed higher Ki67 expression and lower Caspase3 expression at 24 h. Under the corresponding conditions, the spheroids were successfully differentiated into osteogenic and adipogenic lineages. AB-MSC spheroids can induce neural-like cells after neurogenic differentiation. Higher expression of osteogenic markers, adipogenic markers, and neurogenic markers (NF-M, NeuN, and GFAP) was found in spheroids than in the monolayer. Spontaneous spheroids exhibited higher stemness than mechanical spheroids did. HIF-1α and HIF-2α were remarkably upregulated in spheroids. After HIF-1/2α-specific inhibition, spheroid formation was significantly reduced. Moreover, the expression of the pluripotency genes was suppressed.

**Conclusions:**

Spontaneous spheroids from AB-MSCs enhance stemness and pluripotency. HIF-1/2α plays an important role in the stemness regulation of spheroids. AB-MSC spheroids exhibit excellent multi-differentiation capability, which may be a potent therapy for craniomaxillofacial tissue regeneration.

**Supplementary Information:**

The online version contains supplementary material available at 10.1186/s40659-023-00421-w.

## Background

Adult stem cell regeneration therapies and clinical trials have attracted attention as curative treatments for many life threatening diseases [1, 2]. Mesenchymal stem cells (MSCs) are currently the most widely used cell sources, and more than 1000 clinical trials have been registered by 2021 [3]. The application of MSCs also has certain limitations, such as a limited number of donor-derived stem cells [4, 5]. In addition, the acquisition of bone marrow MSCs is accompanied by greater invasiveness, and the cells are susceptible to contamination or loss of stemness in traditional culture processes [6]. The differentiation capacity of MSCs is relatively limited compared to that of pluripotent stem cells. Therefore, maintenance of pluripotent stem cells in vitro plays an important role in tissue regeneration.

Three-dimensional (3D) cell culture methods, including 3D scaffolds, microcarrier cultures, neurospheres, and cellular spheroids, are increasingly used [7–9]. Compared with monolayer cultures, 3D cell cultures can mimic stem cells in vivo and maintain stemness, and are regarded as novel stem cell culture methods [10]. We have recently developed a novel spheroid culture technique for somatic stem cells using a low-adherence specific culture plate [11]. In simple terms, the cells partially attach to the dish, but potent stem cells or progenitor cells divide on the dish and form spheroids, which eventually detach from it. Spontaneous spheroid formation occurs under static conditions. For other spheroid formation methods (e.g., hanging droplets, magnetic levitation, and exclusively non-adherent conditions), various types of cells are forced to aggregate into spheroids. The cell-to-cell attachment is achieved by physical forces, and these are designated as “Mechanical Spheroid Formation” methods [12, 13]. However, the fundamental differences between the spontaneous and mechanical spheroids have yet to be shown. Compared to mechanical spheroids, the spontaneous spheroids ideally consist of a purer stem cell population because spheroid formation starts only from possible stem cells. Spontaneous spheroids can be generated from two-dimensional (2D) cultured oral mucosa, skin, or cortical bone cells and have been proven to contain and maintain high pluripotency [14, 15]. Moreover, spontaneous spheroid formation is efficient, has high yield, and can be cryopreserved, showing great potential for tissue engineering. However, the detailed characteristics of spontaneous spheroids from alveolar bone-derived cells have not been reported.

The alveolar bone is the supporting tissue in the oral cavity, and its remodeling is active because of physiological and functional stimulation [16]. Alveolar bone MSCs are a special type of adult stem cells that originate from the ectoderm during embryonic development and are derived from neural crest stem cells [17]. Therefore, compared with the other bone tissue-derived MSCs, which develop from the mesoderm, alveolar bone MSCs have partially different properties, such as active immunomodulatory effects [18, 19]. Studies have pointed out that alveolar bone MSCs have multi-differentiation and bone regeneration abilities in vivo and can even be obtained from older adults [20–22]. Alveolar bone is more accessible and cost-effective than bone marrow. Dentists can obtain a larger amount of alveolar bone tissue during treatment with less trauma and rapid healing. In this study, we focus on spontaneous spheroids derived from alveolar bone-derived mesenchymal stromal cells (AB-MSCs) and characterize their potential as a superior source of adult stem cells.

The mechanism of stemness acquisition in spheroids is unclear. Hypoxia-inducible factors (HIFs) play a significant role in the maintenance of stemness in embryonic stem cells (ESCs) and cancer stem cells [23, 24]. Moreover, HIFs can regulate the expression of multipotency markers, such as Sox2 and Oct4, in cancer stem cells [25, 26]. The hypoxic environment gradually induces the expression of HIFs during spheroid formation from 2D cultured cells. However, whether HIFs play a key role in stemness acquisition and maintenance of somatic stem cells is unclear.

The objectives of this study are to analyze the pluripotency and multi-differentiation ability of spontaneous spheroids derived from AB-MSCs; compare the stemness between spontaneous spheroids and mechanical spheroids; and describe the effect of HIFs on the stemness maintenance of AB-MSC spheroids.

## Methods

### Cell culture

The animal experiments were approved by the Animal Research Committee of Zhongshan Hospital, Fudan University, Shanghai, China (2016–128). Alveolar bone tissue was obtained from C57/BL6J mice (six weeks old). The protocol was based on a previous study with minor modifications [27]. Mice were euthanized by anesthetic overdose, and soft tissue was separated in the oral cavity with a scalpel until the mandible was exposed. After the mandible was removed, the teeth were extracted and soft tissue was scraped with a curette. The obtained mandible was digested with 0.25% collagenase (FUJIFILM Wako Pure Chemical Corporation, Osaka, Japan) for 15 min and then cut into 1–2 mm pieces with scissors. The tissue fragments were transferred into a digestion solution (0.25% collagenase and 20% fetal bovine serum [FBS; Sigma-Aldrich, Darmstadt, Germany]) for 45 min at 37 °C. The cells were collected into a 50 mL tube using a 40 μm cell strainer (Falcon; Corning, NY, USA), and the supernatant was removed by centrifugation. The harvested cells were seeded in 30 mm dishes (Falcon) for primary culture. The remaining bone fragments were collected and placed in culture dishes for explant culturing. The cells extending from the bone fragments were collected for experiments. The culture medium consisted of α-MEM (HyClone; Fisher, Logan, UT, USA), 10% FBS, 1% penicillin–streptomycin-amphotericin solution (Biological Industries, Cromwell, CT, USA), and 10 ng/mL recombinant human basic fibroblast growth factor (bFGF; Gibco, Carlsbad, CA, USA). The AB-MSCs were cultured in an incubator at 37 °C and 5% CO2. The medium was changed every three days and passaged when 80% confluency was reached.

The bone marrow MSC collection protocol was consistent with that in our previous study [15]. Briefly, the femurs and tibiae were separated cleanly, and the bone marrow was flushed out using a syringe with a 27G needle. The cell suspension was then centrifuged and seeded in a culture dish containing the same culture medium.

### Spontaneous spheroid formation

Passage 2 and 3 cells were obtained to form spontaneous spheroids. As described in a previous study [14], AB-MSCs were seeded into low-adherence specific culture plates (1.5 × 104 cells/cm2) (Azunol, #1–8549-02, AS ONE, Osaka, Japan) to form spheroids. Half of the culture medium was changed every three days. Morphological observation of spheroids was performed using a phase-contrast microscope (Olympus CKX53 inverted microscope, Tokyo, Japan). The number and diameter of spheroids were recorded. Spontaneous spheroid were recovered at 24, 72, and 120 h.

### Mechanical spheroid formation

The hanging droplet method was used to form mechanical spheroids. The AB-MSCs were digested using 0.25% trypsin–EDTA (Gibco, Life Technologies, Carlsbad, CA, USA). Cell suspensions were prepared at a density of 100,000 cells/mL. Using an 8-channel pipette set to 10 µL, approximately 100 drops were transferred to the lid of a 10 cm culture dish (Falcon). When finished, the lid was placed back on the culture dish, so that the droplet could be inverted. Each droplet contained 1000 cells. Cells were observed to aggregate into spheroids after 24 h and then harvested for experiments.

### Cell viability

Cell viability was assessed using a Cell Counting Kit-8 assay (CCK-8; DOJINDO, Shanghai, China). Spheroids and monolayer cultured cells were investigated at 24, 48, 72, 96, and 120 h of culture. Cells were seeded at a density of 5000 cells per well in 96-well plates. Cells were incubated with 10 µl of CCK-8 reagent for 2 h at 37 °C. The result was measured using a Synergy™ HTX Microplate Reader (BioTek Instruments, WINOOSKI, VT, USA) at 450 nm.

### Multi-differentiation capability

#### Osteogenic differentiation

AB-MSC spheroids were harvested after 24 h for osteogenic differentiation. Spheroids or monolayer cultured cells were seeded in 6-well plates (Falcon). When cells reached 70–80% confluency, the culture medium was changed to the osteogenic differentiation solution (culture medium supplemented with 100 nM dexamethasone, 50 µM L-ascorbic acid phosphate, and 10 mM β-glycerophosphate [all from Sigma-Aldrich]) for induction. Bone marrow MSCs were also subjected to osteogenic differentiation, and the differences between AB-MSC spheroids and bone marrow MSCs were compared. Osteogenic induction lasted for 14 d, and the medium was changed every 2 d.

#### Adipogenic differentiation

Spheroids or monolayer cultured cells were seeded into 6-well plates for adipogenic induction. The adipogenic induction solution was culture medium supplemented with 1 µM dexamethasone, 0.5 mM 3-isobutyl-1-methylxanthine (Wako), and 10 µg/mL insulin (Sigma-Aldrich). Adipogenic induction lasted for 10 d, and the medium was changed every 3 d.

#### Neurogenic differentiation

The neurogenic differentiation protocol was the same as that described in our previous study [14]. Spheroids or monolayer cultured cells were induced for 14 d, and 50% of the medium was changed every 2 d.

### Alkaline phosphatase staining and activity assay

An alkaline phosphatase (ALP) staining kit (Beyotime Institute of Biotechnology, Shanghai, China) was used to evaluate ALP activity after osteogenic induction, according to the manufacturer’s protocol. ALP-positive cells were observed using a phase-contrast microscope (Olympus CKX53 inverted microscope). Meanwhile, an ALP activity assay was performed to evaluate osteogenic capacity. ALP activity was determined using an ALP assay kit (Sigma-Aldrich) and absorbance was measured at 405 nm. The ALP activity was normalized to the total protein content of each sample.

### Oil-red O staining

After adipogenic induction, the cells were fixed with 4% paraformaldehyde phosphate buffer solution (Wako) for 15 min and then treated with Oil Red O working solution (Shanghai LMAI Bio, Shanghai, China) for 10 min. The lipid droplets were observed using a phase-contrast microscope (Olympus CKX53 inverted microscope).

### Immunofluorescence staining

Immunofluorescence staining was performed to confirm the pluripotency of AB-MSC spheroids. This protocol was consistent with that reported previously [14]. In brief, spheroids were collected and solidified in iPGell (Genostaff, Tokyo, Japan) according to the manufacturer’s protocol. The samples were fixed with 4% paraformaldehyde in phosphate buffer (Wako), embedded in paraffin, and sectioned at a thickness of 8 μm. Primary antibodies for pluripotency markers included Oct4 (ab19857), Sox2 (ab79351), KLF4 (ab216875), and cMyc (ab32072). The primary antibodies for neuronal markers were neurofilament medium (NF-M, ab7794), βIII tubulin (ab78078), NeuN (ab12763), and GFAP (ab279290). The cell proliferation marker Ki67 (ab15580) and apoptosis marker Caspase3 (ab13585) were also analyzed. The secondary antibodies were goat anti-rabbit IgG (Alexa Fluor 647, ab150079) and goat anti-mouse IgG (Alexa Fluor 488, ab150113). Nuclei were stained with 4',6-diamidino-2-phenylindole (DAPI, ab104139). All antibodies were purchased from Abcam (Cambridge, UK). Positive fluorescent staining was observed using an inverted microscope (Olympus IXplore Spin microscope; Tokyo, Japan).

### Reverse transcription quantitative polymerase chain reaction

Total RNA was extracted using TRIzol reagent (Invitrogen, Carlsbad, CA, USA). cDNA was synthesized using a PrimeScript RT kit (TaKaRa, Kusatsu, Japan) according to the manufacturer’s protocol. The SYBR Green qPCR assay kit (TaKaRa) was used for Reverse transcription quantitative polymerase chain reaction (RT-qPCR) analysis. Gene expression was calculated by using the ∆∆Ct method, GAPDH was used as a housekeeping gene, and the experiment was repeated at least three times. The primers used for RT-qPCR are listed in Table 1.

**Table 1:** primer sequences for PCR

Gene	Forward primers (5′-3′)	Reverse primers (5′-3′)
GAPDH	AACTTTGGCATTGTGGAAGG	ACACATTGGGGGTAGGAACA
CD51	AAGAGTTTGTTGCCGCCTTA	CAATGGCCACACACAGAGAC
CD29	AACTCCGACGCCTTTTCTTT	CCCCCATATTGCAAACAGAC
CD105	CTTCCAAGGACAGCCAAGAG	GTGGTTGCCATTCAAGTGTG
Sca1	CTGTGGTGGGGTGCTTTACT	GCCAGTGCAGAAGGGTAGAG
Ki67	AAGAGCAGGTTAGCACTGTTATGAA	TGCAGATGCATCAAACTTGG
Caspase3	GGGCCTGTTGAACTGAAAA	CCGTCCTTTGAATTTCTCCA
Oct4	CAGACCACCATCTGTCGCTTC	AGACTCCACCTCACACGGTTCTC
SSEA1	GCAGGGCCCAAGATTACTGAC	AAGCGCCTGGGCCTAAGAA
KLF4	AACATGCCCGGACTTACAAA	TTCAAGGGAATCCTGGTCTTC
Sox2	GTTCTAAGTGGTACGTTAGGCGCTTC	TCGCCCGGAGTCTAGCTCTAAATA
cMyc	ACCCTCAAACTCCTGGTCCT	CAGGATGTAGGCGGTGGCTT
Nanog	AACATGCCCGGACTTACAAA	ACCCTCAAACTCCTGGTCCT
OPG	CTGCCTGGGAAGAAGATCAG	TTGTAAGCTGTGCAGGAAC
Runx2	CCCAGCCACCTTTACCTACA	TATGGAGTGCTGCTGGTCTG
DMP1	AGTGAGTCATCAGAAGAAAGTCAAGC	CTATACTGGCCTCTGTCGTAGCC
BSP	GAGACGGCGATAGTTCC	AGTGCCGCTAACTCAA
PPARG	GACAGGCATGGAAATGGAGT	CAGAAATCCTCCTGCCTCTG
LPL	GGGCTCTGCCTGAGTTGTAG	CCATCCTCAGTCCCAGAAAA
NF-M	GCCGAGCAGACCAAGGAGGCCATT	CTGGATGGTGTCCTGGTAGCTGCT
NeuN	GTAGCGGTCCATACCAGGAA	CAGCCTGACTTCAGGGACTC
GFAP	ACCTCGGCACCCTGAGGCAG	CCAGCGACTCAACCTTCCTC
HIF-1α	TCAAGTCAGCAACGTGGAG	TATCGAGGCTGTGTCGACTG
HIF-2α	CAGTACTCCCACAGGCCTGACTAAC	GACTGTCACACCGCTGCCATA
VEGF	GCTCTCCACGATTTGACCAT	ATCCACCCACTAGGCAACAG

### HIF-1a or HIF-2a inhibition

The HIF-1 specific inhibitor topotecan (#123948–87-8, Sigma-Aldrich) and the HIF-2 specific inhibitor PT2385 (#1672665–49-4, Selleck, Shanghai, China) were used to block the mRNA expression of HIFs. Working concentrations were obtained from previous studies [28, 29], and cell viability assays (Cell Counting Kit-8, CCK-8, Apexbio, Houston, TX, USA) were also performed to identify the appropriate concentrations. The inhibitors were diluted in dimethyl sulfoxide (DMSO) and stored at − 80 °C.

### Statistical analysis

Statistical analysis was performed using the SPSS16.0 software (SPSS Inc, Chicago, IL, USA). The results are expressed as mean ± standard deviation (SD). The Student’s t-test was used to compare two groups. The statistical significance between multiple groups was compared using one-way analysis of variance (ANOVA) followed by Dunnett's test. Statistical significance was set as p < 0.05.

## Results

### Spontaneous spheroid formation from AB-MSCs

**Fig. 1:**
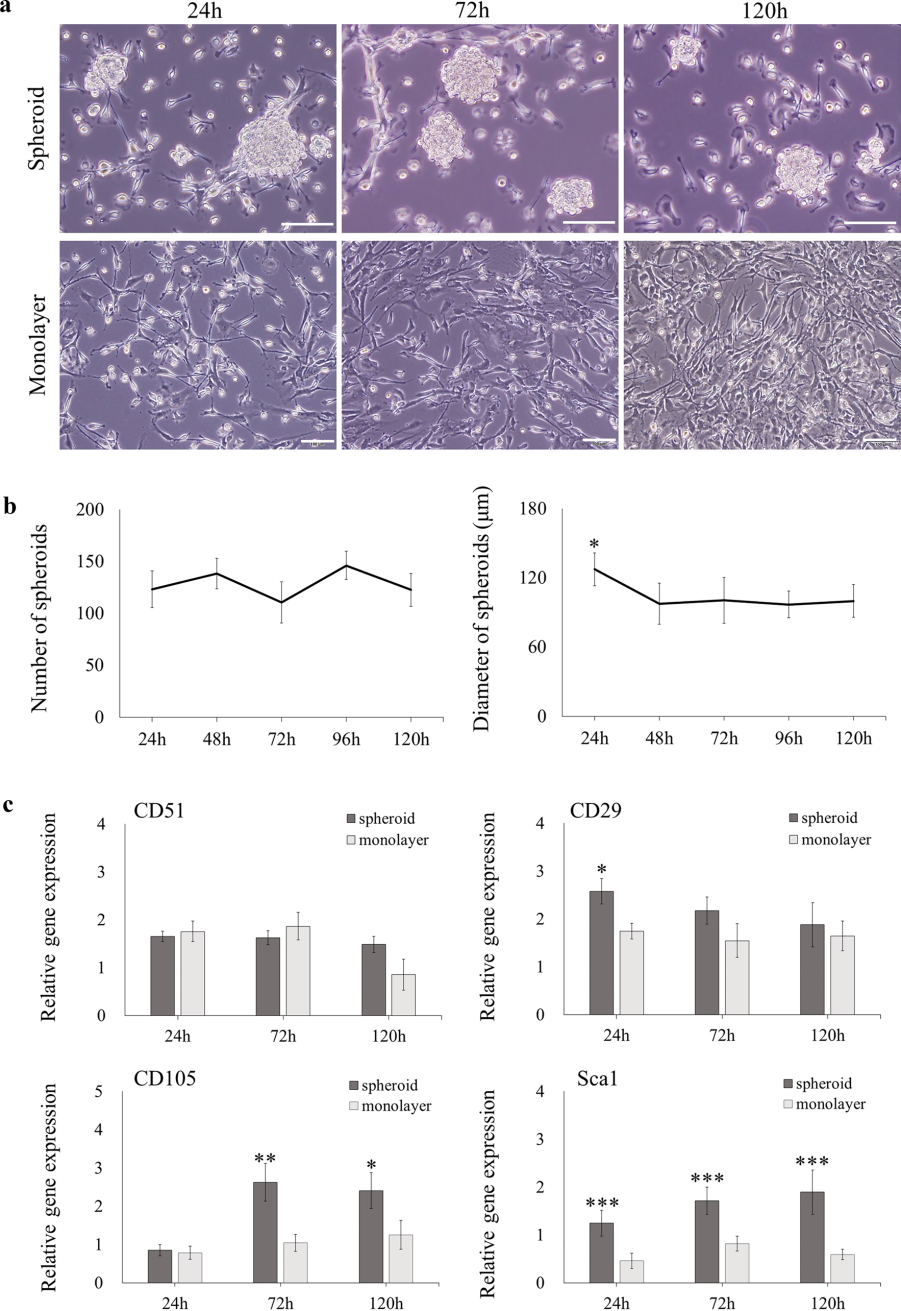
AB-MSCs formed spontaneous spheroids. **a** The morphology of AB-MSC spheroids and monolayer was observed during 120 h culture. Scale bars = 100 μm. **b** The number and diameter of spheroids were tested (n = 20, *, p < 0.05, ANOVA test). **c** The expression of MSC markers CD51, CD29, CD105, and Sca1 in spheroids and monolayer was analyzed via RT-qPCR (n = 3, *, p < 0.05; **, p < 0.01; ***, p < 0.001, Student’s t-test, compared to the monolayer)

The AB-MSCs spontaneously formed spheroids after 24 h of culturing. Morphological observations revealed that spheroids remained spherical during the culture period, while monolayer cells were spindle-shaped (Fig. 1a). The number of spheroids was relatively stable during 5 d of culture. The diameter of the spheroids was the highest at 24 h, with a statistically significant difference (p < 0.05) and then decreased slightly. The average diameter of AB-MSC spheroids was 104.39 μm (Fig. 1b). The RT-qPCR results showed that AB-MSC spheroids were positive for the MSC markers CD51, CD29, CD105, and Sca1 (Fig. 1c). The expression of Sca1 was significantly higher in spheroids than in monolayer cultures (p < 0.001).

### Cell proliferation and apoptosis analysis in AB-MSC spheroids

**Fig. 2:**
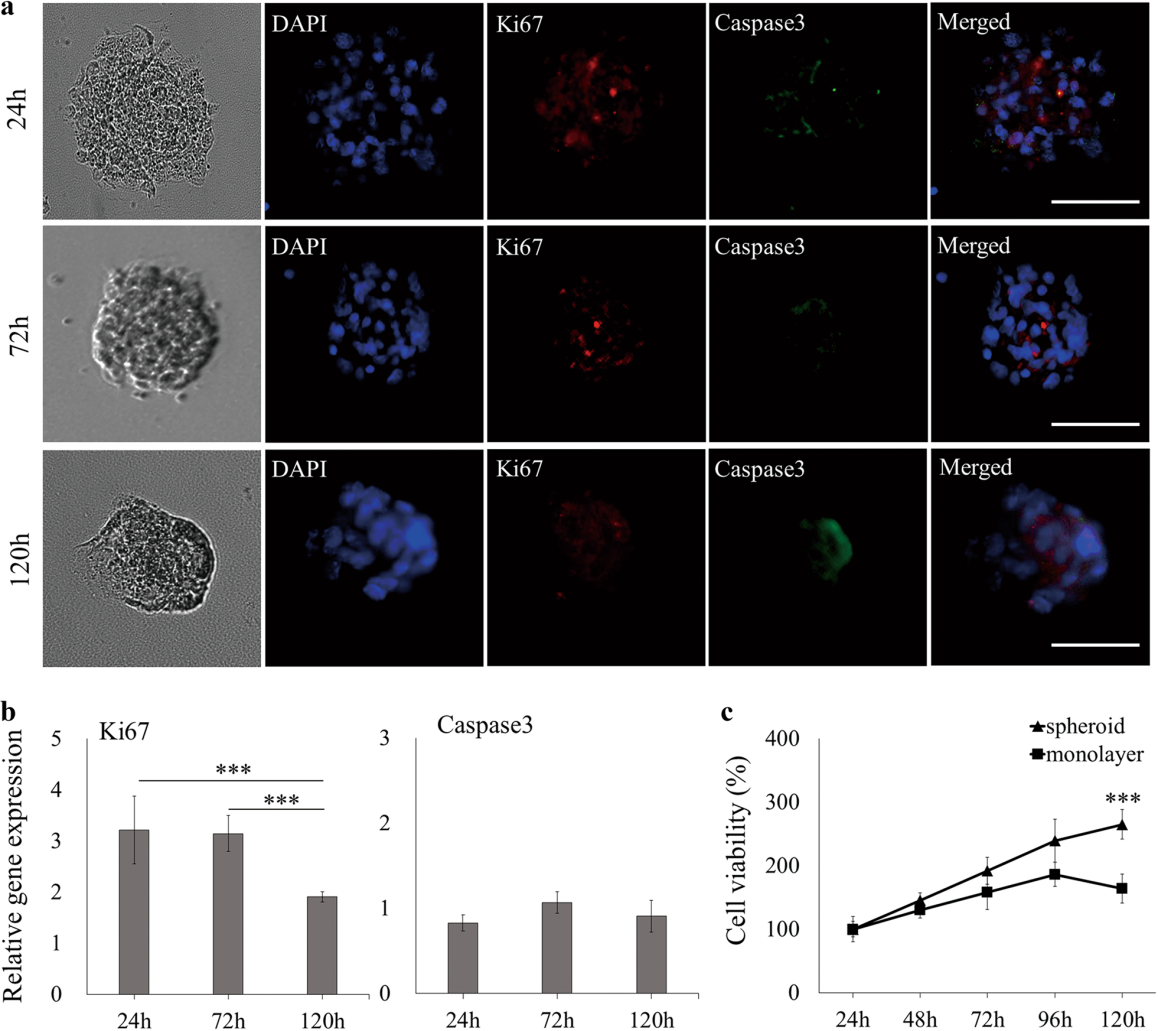
Analysis of cell proliferation and apoptosis in AB-MSC spheroids. **a** Immunofluorescence was used to assess the expression of cell proliferation marker Ki67 (red) and apoptosis marker Caspase3 (green) in spheroids. Scale bars = 100 μm. DAPI, 4',6-diamidino-2- phenylindole. **b** RT-qPCR analysis of Ki67 and Caspase3 expression in spheroids during 120 h culture (n = 3, ***, p < 0.001, ANOVA test). **c** Cell viability of spheroids and monolayer was measured by CCK-8 (n = 3, ***, p < 0.001, Student’s t-test, compared to the monolayer)

To assess the viability of AB-MSC spheroids, the expression of the cell proliferation marker Ki67 and apoptosis marker Caspase3 was analyzed. Immunofluorescence showed that most cells in the spheroids were positive for Ki67, and the expression was most obvious at 24 h. Only a very small number of cells showed Caspase3-positive expression (Fig. 2a). The RT-qPCR results also supported this observation. The expression of Ki67 was significantly higher in 24 and 72 h spheroids (p < 0.001). However, no difference was observed in the expression of Caspase3 (Fig. 2b). In addition, CCK-8 assay showed that the spheroids maintained high cell viability during the observation period. Compared with monolayer cultured cells, spheroids culture conditions significantly maintained the viability at 120 h (Fig. 2c).

### Pluripotency analysis of AB-MSC spheroids

**Fig. 3:**
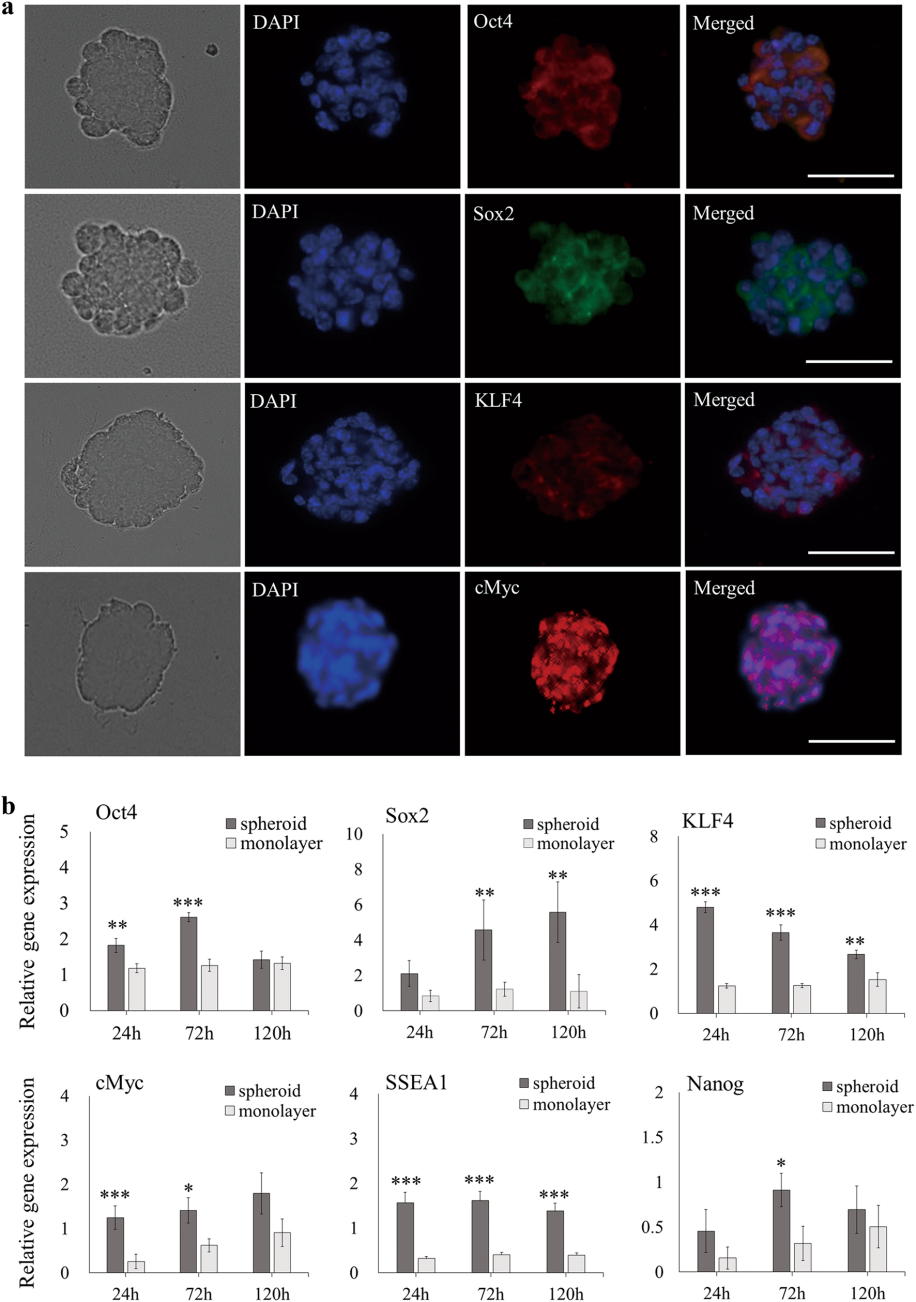
Characterization of pluripotency in AB-MSC spheroids. **a** The expression of pluripotency markers Oct4 (red), Sox2 (green), KLF4 (red), and cMyc (red) in spheroids was evaluated via immunofluorescence. Scale bars = 100 μm. DAPI, 4',6-diamidino-2- phenylindole. **b** The expression of pluripotency markers and ESC markers (SSEA1 and Nanog) in spheroids and monolayer was analyzed via RT-qPCR (n = 3). *, p < 0.05; **, p < 0.01; ***, p < 0.001, Student’s t-test, compared to the monolayer

To evaluate stemness in AB-MSC spheroids, pluripotency markers Oct4, Sox2, KLF4, and cMyc were stained. Immunofluorescence showed strong positive staining in 24 h spheroids (Fig. 3a). Pluripotency markers and ESC markers (SSEA1 and Nanog) expression in spheroids and monolayers was analyzed using RT-qPCR. The expression of Oct4 and cMyc was higher in spheroids at 24 and 72 h (p < 0. 05). The expression of Sox2 did not differ at 24 h, but its expression in spheroids increased significantly at 72 and 120 h. During the culture period, the expression of both KLF4 and SSEA1 was significantly upregulated in spheroids compared to that in the monolayer (p < 0. 01). The expression of Nanog was significantly higher in spheroids at 72 h (Fig. 3b).

### Evaluation of the multi-differentiation ability of AB-MSC spheroids

**Fig. 4:**
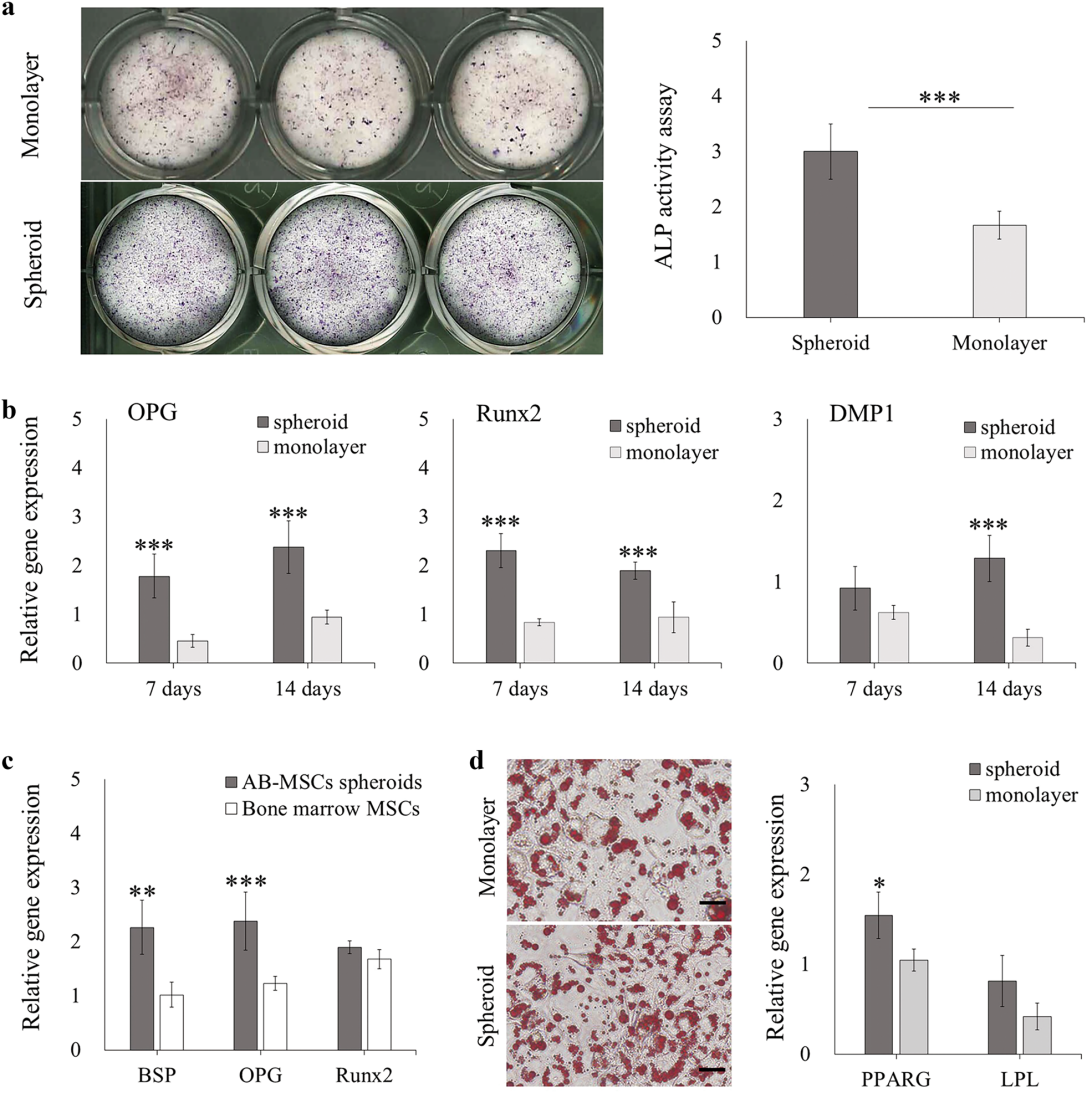
Osteogenic and adipogenic capability of AB-MSC spheroids. **a** After 14 d of osteogenic induction, ALP staining and ALP activity assay were used to evaluate ALP activity in spheroids and monolayer (n = 3). **b** The expression of osteogenic markers was analyzed via RT-qPCR on days 7 and 14 of osteogenic induction (n = 3). **c** Differences in osteogenic capacity between AB-MSC spheroids and bone marrow MSCs were compared by using RT-qPCR after 14 d of osteogenic differentiation (n = 3). **d** Oil-red O staining was used to assess lipid droplets after 10 d of adipogenic induction in spheroids and monolayers. The expression of adipogenic markers was analyzed via RT-qPCR (n = 3). Scale bars = 100 μm. *, p < 0.05; **, p < 0.01; ***, p < 0.001, Student’s t-test, compared to the monolayer

The AB-MSC spheroids and monolayers were subjected to osteogenic differentiation. ALP staining was performed 14 d after induction, and the results showed that the number of positively stained cells was higher in the spheroids. The ALP assay also showed that ALP activity in spheroids was significantly higher than that in monolayer cultures (Fig. 4a). The expression of osteogenic markers was analyzed by using RT-qPCR after 7 and 14 d of osteogenic induction. The mRNA expression of OPG and Runx2 was remarkably high in the spheroids (p < 0.001). Although DMP1 was indistinguishable on day 7, it was significantly higher than that of the monolayer after 14 d (Fig. 4b). We also compared the differences in osteogenic capacity between AB-MSC spheroids and bone marrow MSCs, which are considered as a superior source for craniofacial bone regeneration [30]. After 14 d of osteogenic induction, the expression of BSP and OPG significantly increased in AB-MSC spheroids (p < 0.01). However, there was no difference in Runx2 (Fig. 4c).

After adipogenic induction of AB-MSC spheroids and monolayers, lipid droplets were successfully observed using oil-red O staining (Fig. 4d). Then, the expression of PPARG and LPL was analyzed using RT-qPCR. The expression of PPARG was higher in spheroid-derived cells than in monolayer cultured cells (Fig. 4d).

**Fig. 5:**
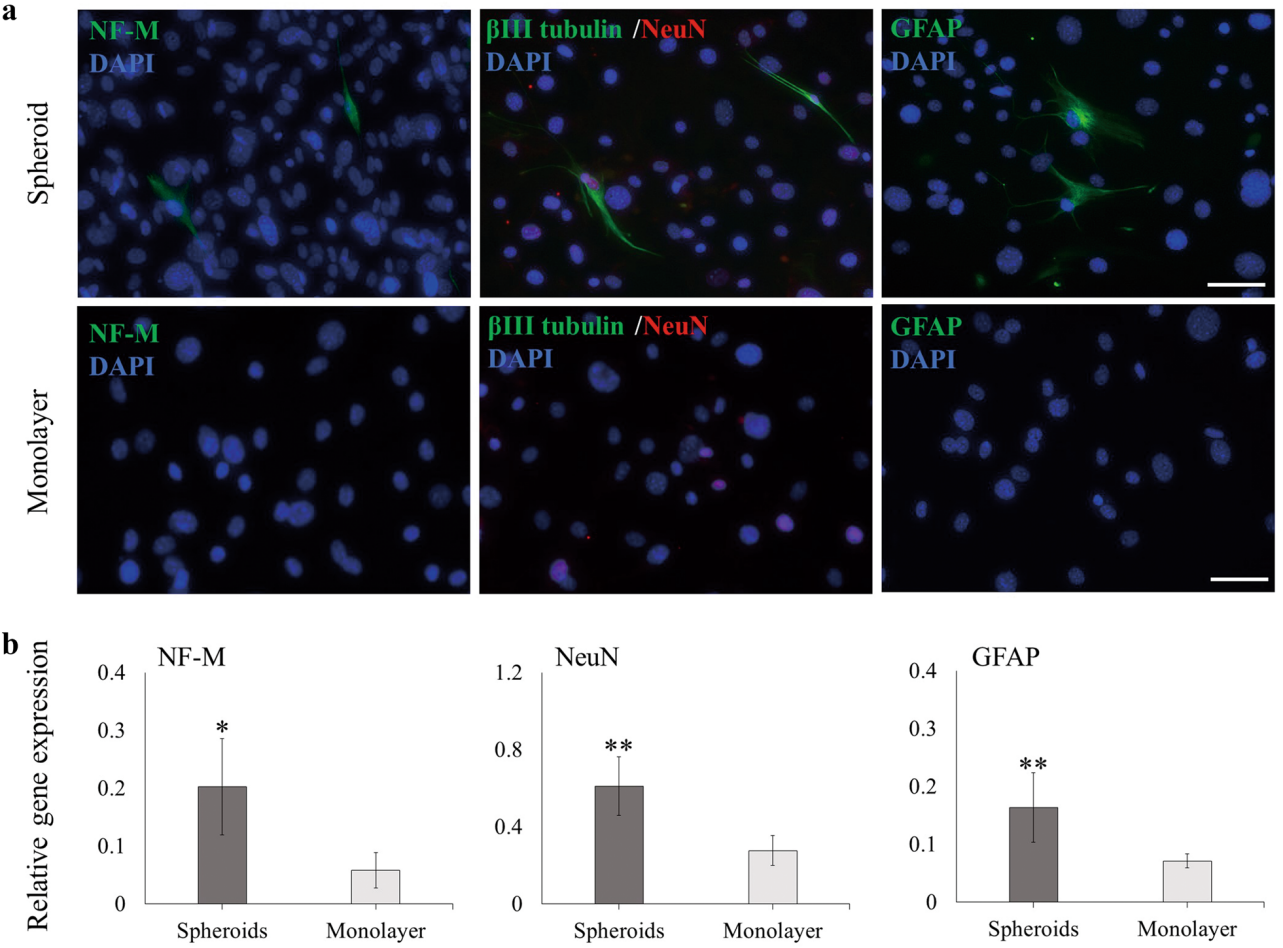
Neurogenic capability of AB-MSC spheroids. **a** Neurogenic differentiation of spheroids and monolayer for 14 d. Immunofluorescence showed positive staining for neurogenic markers NF-M (green), βIII tubulin (green), NeuN (red), and GFAP (green) in spheroid-derived cells. Scale bars = 50 μm. DAPI, 4',6-diamidino-2- phenylindole. (**b**) The RT-qPCR analysis of mRNA expression levels of NF-M, NeuN, and GFAP in spheroids and monolayer (n = 3). *, p < 0.05; **, p < 0.01, Student’s t-test, compared to the monolayer

The spheroids and monolayer cells were subjected to neurogenic differentiation for 14 d. Immunofluorescence showed that a small number of neural-like cells were successfully induced in the spheroid-derived cells (Fig. 5a). After induction, spheroid-derived cells were stained positively for neurogenic markers NF-M, βIII tubulin, NeuN, and GFAP, and the ratio of positive cells was 0.17%, 0.36%, 1.25%, and 0.24%, respectively. Monolayer cells contained some cells positive for NeuN, but they were negative for NF-M and GFAP (Fig. 5a). Furthermore, the mRNA expression levels of NF-M, NeuN, and GFAP were significantly higher in spheroid-derived cells than in monolayer cells (Fig. 5b).

### Stemness differences between spontaneous and mechanical spheroids

**Fig. 6:**
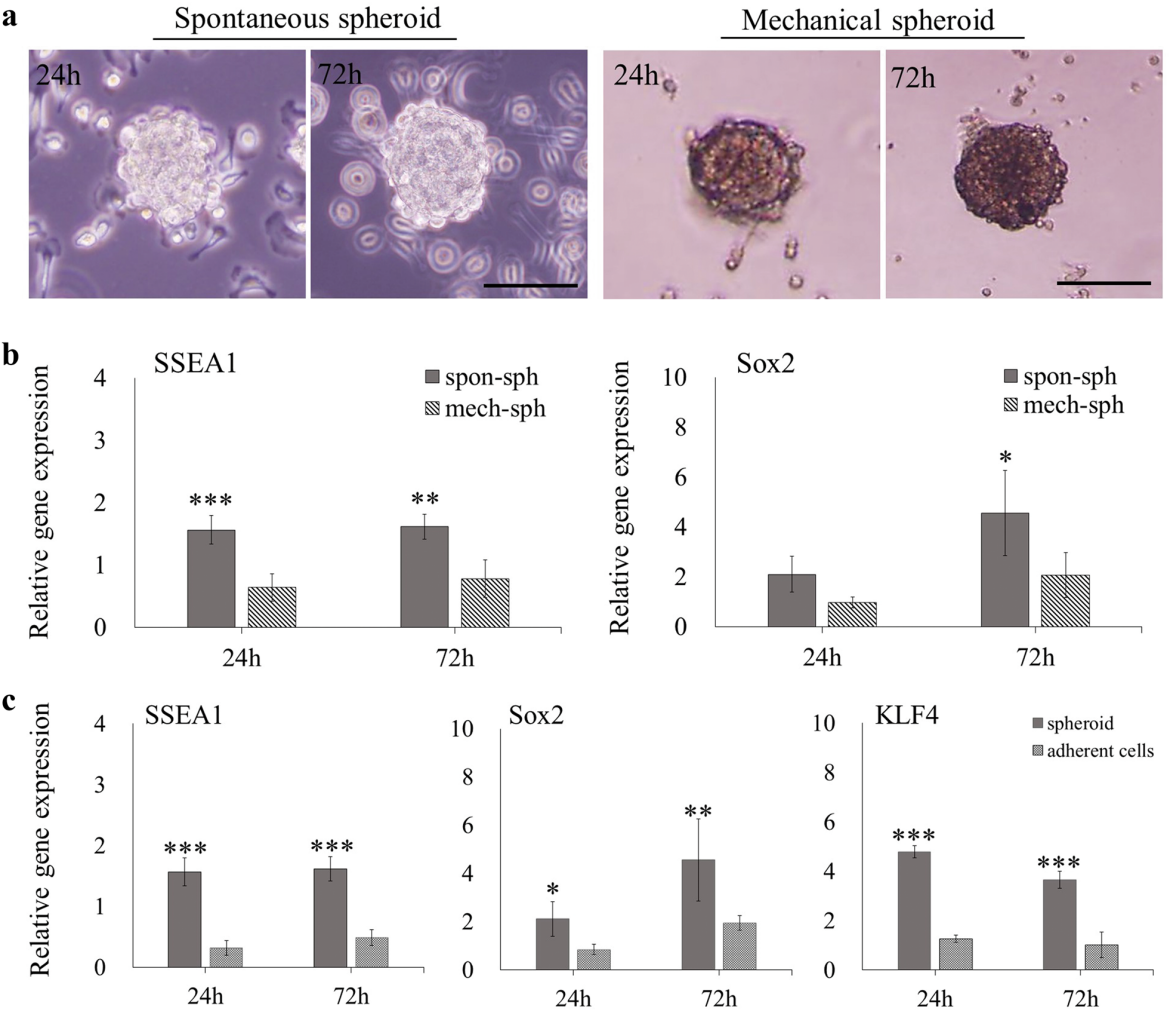
Comparison of stemness between spontaneous and mechanical spheroids. **a** Morphological observation of spontaneous and mechanical spheroids (1000 cells) at 24 and 72 h. Scale bars = 100 μm. **b** The mRNA expression levels were analyzed between spontaneous and mechanical spheroids (n = 3). **c** RT-qPCR analysis of the expression of pluripotency markers in floating spheroids and adherent cells in low-adherence specific culture plates (n = 3). *, p < 0.05; **, p < 0.01; ***, p < 0.001, Student’s t-test, compared to mechanical spheroids or the adherent cells

The average diameter of the AB-MSC spontaneous spheroids was 104.39 μm. To compare the spheroids formed by using the two methods, mechanical spheroids with similar diameters were selected. A total of 1000 AB-MSCs formed mechanical spheroids using the hanging droplet method, and the average diameter was measured to be 106.54 μm. Morphological observations showed that the spontaneous spheroids could detach from the culture dish and float after maturation, and some adherent cells were still attached to the dish. However, in mechanical spheroids, all cells aggregated into spheroids (Fig. 6a). The RT-qPCR results showed that SSEA1 mRNA expression levels were remarkably higher in spontaneous spheroids. The expression of Sox2 in spontaneous spheroids was significantly higher than that in mechanical spheroids at 72 h (Fig. 6b).

We compared the adherent cells and spheroids in a spontaneous spheroid culture system. The expression of SSEA1, Sox2, and KLF4 was significantly higher in floating spheroids than in adherent cells on specific culture plates (Fig. 6c).

### Effect of HIF-1/2α on stemness maintenance of AB-MSC spheroids

**Fig. 7:**
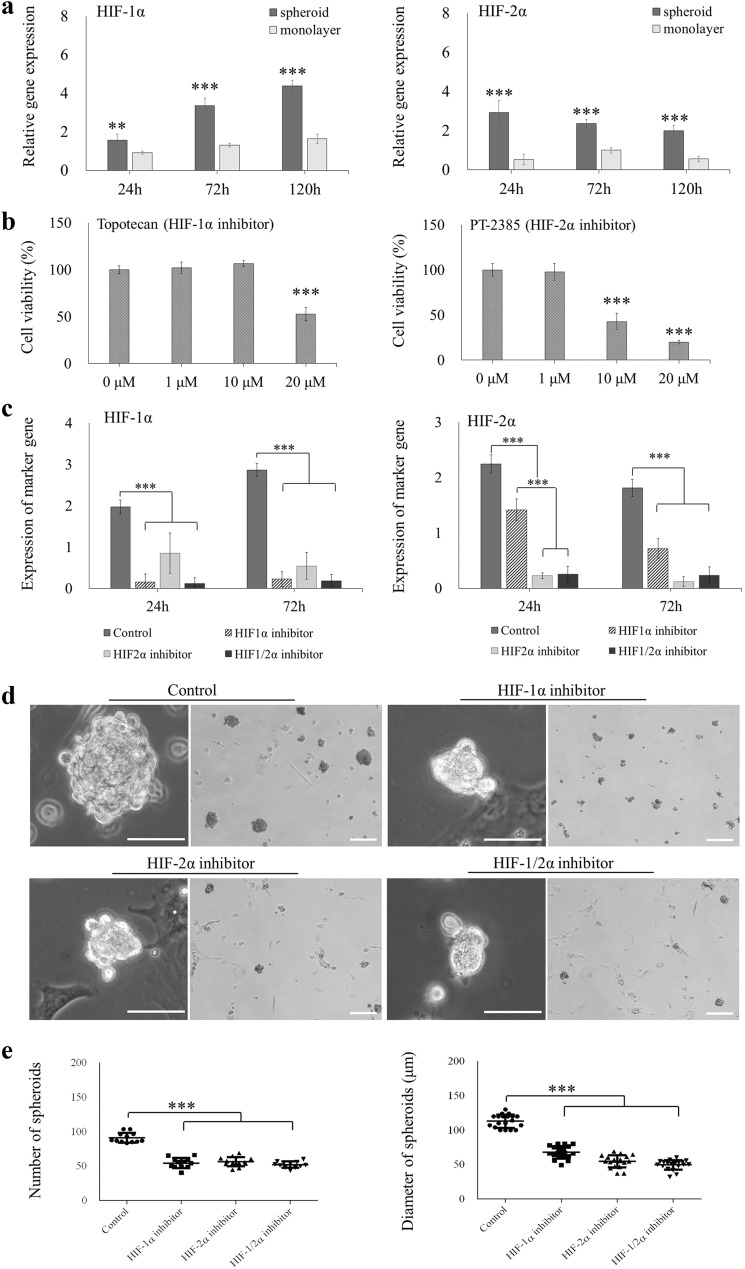
Effect of HIF-1/2α inhibition on AB-MSC spheroids formation. **a** Analysis of HIF-1α and HIF-2α expression in spheroids and monolayer by RT-qPCR (n = 3, **, p < 0.01; ***, p < 0.001, Student’s t-test, compared to the monolayer). **b** CCK-8 assay was used to assess cell viability under treatment with different concentrations of HIF-1/2α specific inhibitor (topotecan) and HIF-2α specific inhibitor (PT-2385) (n = 3, ***, p < 0.001, ANOVA test). **c** RT-qPCR was used to assess the expression of HIF-1/2α following HIFs inhibitor treatment (n = 3, ***, p < 0.001, ANOVA test). **d** After HIF-1/2α inhibitors treatment, the formation of spheroids was observed at 24 h. Scale bars = 100 μm. **e** The number and diameter of spheroids were evaluated after HIFs inhibition (n = 20, ***, p < 0.001, ANOVA test)

The expression levels of HIF-1α and HIF-2α were significantly higher in spontaneous spheroids than in monolayer cells (Fig. 7a). The CCK-8 assay was used to determine the working concentrations of the inhibitors (Fig. 7b). Although 10 μM topotecan (HIF-1α inhibitor) and 1 μM PT-2385 (HIF-2α inhibitor) had no effect on cell viability, the RT-qPCR results showed that HIF-1α and HIF-2α mRNA expression were successfully suppressed after treatment with the inhibitors (Fig. 7c). Moreover, HIF-1α or HIF-2α inhibitors severely affected spheroid formation of AB-MSCs at 24 h (Fig. 7d). The number of spheroids was remarkably reduced after inhibition of HIFs compared to the control (p < 0.001). Similarly, the diameter of the spheroids also significantly decreased after inhibition of HIFs. The smallest cell diameter was observed for HIF-1/2α co-inhibition (Fig. 7e).

**Fig. 8:**
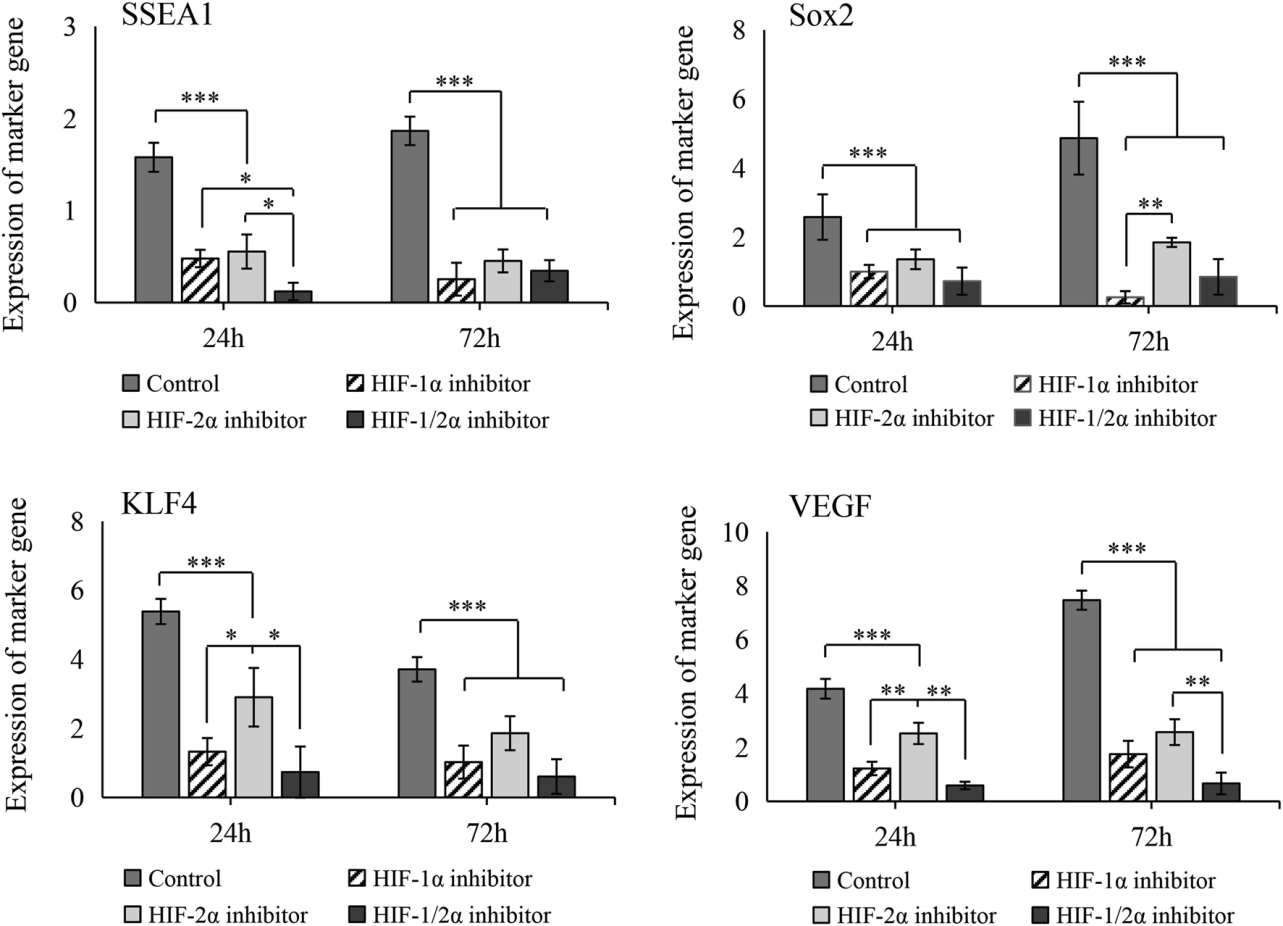
Effect of HIF-1/2α inhibition on stemness of AB-MSC spheroids. Expression levels of pluripotency markers in spheroids were analyzed by using RT-qPCR after HIFs inhibitor treatment (n = 3). *, p < 0.05; **, p < 0.01; ***, p < 0.001, ANOVA test

To investigate the role of HIFs in AB-MSC spheroid stemness maintenance, pluripotency markers and VEGF expression levels were analyzed using RT-qPCR. The expression level of SSEA1 significantly decreased after HIF-1/2α inhibition, and the inhibition effect was the greatest after 24 h of co-inhibition. The mRNA expression of Sox2 was also significantly inhibited, and HIF-1α inhibition was most pronounced at 72 h. KLF4 and VEGF expression was attenuated after inhibition, and the inhibitory effect of HIF-1α was more significant than that of HIF-2α at 24 h (Fig. 8).

## Discussion

### Pluripotency characteristics of AB-MSC spheroids

The AB-MSCs were successfully isolated and cultured. The results confirmed that AB-MSCs express the MSC markers CD51, CD29, CD105 and Sca1, and negative for CD45, which is consistent with the MSCs derived from other tissues (Additional file 1: Figure S1) [31]. The AB-MSCs were able to form spontaneous spheroids in low-adherence specific culture plates. Spheroids formed within 24 h, followed by stable maintenance. The diameters of the spheroids were also relatively uniform. The cell proliferation marker Ki67 was highly expressed in spheroids, whereas apoptotic cells were rare, indicating that cells inside the spheroids were healthy. Furthermore, we observed that Sca1 expression was higher in spheroids than in monolayers. Sca1 plays an important role in stem/progenitor cell self-renewal and differentiation [32]. This result indicates that spheroid formation further enhances stemness.

We analyzed the expression of pluripotency markers in AB-MSC spheroids. Both immunofluorescence and RT-qPCR results showed markedly high expression of Oct4, SSEA1, Sox2, KLF4, and cMyc in the AB-MSC spheroids. Among them, the expression of Oct4 significantly increased at 24 and 72 h and then decreased. Oct4 plays a significant role in the early stage of reprogramming and is considered a promoter that regulates metabolic processes [33]. Furthermore, endogenous activation of Oct4 can occur in partially reprogrammed cells. Conversely, the expression of Sox2 increased over time in spheroids. This tendency was the same for spheroids derived from oral mucosa-derived cells [14]. Sox2 is normally activated during the later stages of reprogramming and can also trigger the expression of a series of pluripotency genes [34]. However, the mechanism underlying stemness acquisition after spontaneous spheroid formation remains unclear.

### Multi-differentiation capacity of AB-MSC spheroids

Alveolar bone MSCs showed superior bone regeneration ability in both in vitro and in vivo experiments [20, 21]. However, studies on bone regeneration of AB-MSC spheroids have rarely been reported. In this study, AB-MSC spheroids exhibited stronger osteogenic differentiation ability than monolayer cells. Moreover, BSP, OPG, and Runx2 mRNA expression levels after osteogenic induction were higher in AB-MSC spheroids than in bone marrow MSCs. AB-MSCs are a unique source of stem cells with specific biological characteristics. The alveolar bone is subject to constant occlusal force, and has an active immune microenvironment [27]. Liu et al. [20] also showed that homeobox genes (HOX) in alveolar bone MSCs were significantly higher than those in bone marrow MSCs, which control the proliferation of MSCs and the development of craniofacial bone. Therefore, AB-MSCs can be a superior source of adult stem cells for tissue regeneration.

A few studies have confirmed that dental tissue-derived stem cells, dental pulp spheroids, and oral mucosa spheroids have neurogenic differentiation capabilities [14, 35]. Similarly, neural induction was performed using AB-MSC spheroids. The results showed that spheroids successfully differentiated into neural-like cells with high expression levels of NF-M, NeuN, and GFAP owing to the origin of AB-MSC during embryonic development. Most oral tissues originate from the neural crest; therefore, AB-MSC spheroids may have characteristics of neural crest-derived stem cells. Alveolar bone tissue is readily available in dental clinics with minimal trauma and low donor morbidity. After spontaneous spheroid culture, AB-MSC spheroids showed excellent osteogenic and neurogenic differentiation potential and might be a potential cell source for the treatment of craniomaxillofacial bone defects and neurodegenerative diseases.

### Advantages of spontaneous spheroids compared to mechanical spheroids

This study is the first to compare the spontaneous and mechanical spheroids of AB-MSCs. The results showed that, during spontaneous spheroids formation, some potent stem or progenitor cells initially start to aggregate, and after spheroids form and compact, they detach from the culture dishes and float. The entire process is performed under static conditions. The mechanical spheroids formation process is relatively “rude.” Not only stem cells but also other type of cells can aggregate into mechanical spheroids, depending on their physical force [36]. Our results showed that the expression of SSEA1 and Sox2 were significantly higher in spontaneous spheroids than in mechanical spheroids. Thus, we hypothesis that spontaneously formed spheroids may have more advantages, since it can achieve a more selective culture of somatic stem cells from the start.

We further analyzed the differences in stemness between the remaining adherent cells and spheroids in the spontaneous spheroid culture system. The results showed that spheroid-forming cells had higher expression of SSEA1, Sox2, and KLF4 than non-spheroid-forming cells. These results also support our hypothesis that spontaneous spheroids are more selective for stem cells. Mechanical spheroid formation methods require specialized equipment, and spheroid aggregates are not uniform or suitable for long-term culture [37]. In contrast, spontaneous spheroid formation is a more feasible, simple process and results in uniform spheroid aggregates. Moreover, spontaneous spheroid formation ability is not affected by cell passage. Thus, the spontaneous spheroid method is appropriate for 3D culture.

### Effect of HIF-1/2α on stemness maintenance in AB-MSC spheroids

AB-MSC spheroids exhibited superior pluripotency and multi-differentiation ability. However, the mechanism by which stemness is maintained in spontaneous spheroids remains unclear. Compared to the monolayer culture, both the cell microenvironment and cell-to-cell contacts changed after spheroid formation. The microenvironment of spheroids is complex, with marked changes in cytoskeletal proteins, adhesive molecules, and the extracellular matrix [38]. Spheroid pluripotency is regulated by multiple pathways. Epigenetic regulation has been shown to modulate pluripotency in MSC spheroids [39]. Jeon et al. [40] showed that MSC spheroids caused changes in histone-modifying enzymes and microRNAs (miR-166, miR-175, and miR-146b), which are key molecules in epithelial-mesenchymal transition (EMT). Li et al. [41] indicated that mechanotransduction may modulate the promotion of reprogramming in keratocyte spheroids.

In this study, we investigated the role of HIF-1/2α in maintaining stemness in AB-MSC spheroids. The expression of HIF-1α and HIF-2α in spheroids was significantly higher than that in monolayers. During spheroid formation and maturation, hypoxic environments are locally induced inside spheroids. Particularly in cancer stem spheroids, a significant gradient of oxygen concentration can be observed, with a high surface oxygen concentration and a hypoxic core inside [42]. HIFs, which are regulated by hypoxia, are key transcription factors that regulate hypoxia-related genes. In AB-MSC spheroids, the expression of HIF-1α increased in a time-dependent manner. HIF-2α expression in the spheroids gradually decreased after 24 h. It is conceivable that the regulation of HIF-2α via several pathways, which may not be completely oxygen-dependent. Further study will be needed to clarify this mechanism. Several studies have also reported high protein expression of HIF-1/2α in compact bone- or dental pulp-derived spheroids [15, 43].

Subsequently, the efficiency of AB-MSC spheroid formation and the expression of pluripotency markers were evaluated using a HIF-specific block. The HIF block severely affected spontaneous spheroid formation, and the expression of SSEA1, SOX2, and KLF4 was significantly suppressed. HIF-1/2α co-inhibition most severely affected the stemness of AB-MSC spheroids. Similar to human ESCs, HIF-2α silencing can reduce the protein expression of Oct4, Sox2, and Nanog [26]. Our results indicate that HIF-1/2α can be upstream genes that regulate the expression of pluripotency markers in AB-MSC spheroids. HIF-1/2α can mediate SOX2 mRNA demethylation to inhibit the proliferation and stemness in cancer stem cells [44]. HIF-1/2α also regulates EMT in cancer stem cells to promote cell progression and metastasis [45]. Therefore, HIF-1/2α high expression in AB-MSC spheroids can modulate EMT regulation, which is associated with epigenetic regulation, and enhanced stem cell niche activity in spheroids. VEGF expression was remarkably inhibited after HIF-1α blockade. HIF-1α promotes angiogenesis by regulating VEGF [46]. Our results showed that the internal hypoxic environment in AB-MSC spheroid could upregulate the proangiogenic ability.

This is the first study to demonstrate the effects of HIFs on the acquisition and maintenance of stemness in spontaneous spheroids. Pluripotency in AB-MSC spheroids can be triggered by the upregulation of HIF-1/2α. However, the mechanism of activation and stabilization of HIFs in spheroids requires further study. The results showed that AB-MSC spheroids showed greater osteogenic capacity than bone marrow MSCs, indicating the potential usefulness of AB-MSCs for tissue regeneration. To further demonstrate the therapeutic superiority of the AB-MSCs spheroids, the cells derived from human will be collected. Human AB-MSCs spheroids for in vivo tissue regeneration and safety should be studied in the future.

## Conclusions

AB-MSCs successfully formed spontaneous spheroids. AB-MSC spheroids exhibited superior pluripotency, and HIF-1/2α played an important role in the stemness regulation of spheroids. The spontaneous spheroid method was more selective for stem cells than the mechanical method. AB-MSC spheroids showed excellent osteogenic and neurogenic differentiation capabilities, which may be a potent therapy for craniomaxillofacial tissue regeneration.

## Supplementary Information

**Additional file 1: Figure S1.** The results from flow cytometry of AB-MSCs for mesenchymal stem cell markers. (a-d) AB-MSCs were positive for CD29, CD51 and Sca-1 and negative for CD45.

## Data Availability

Contact to the corresponding author for availability.

## Abbreviations

3D: Three-dimensional
2D: Two-dimensional
AB-MSCs: Alveolar bone-derived mesenchymal stromal cells
RT-qPCR: Reverse transcription quantitative polymerase chain reaction
ALP: Alkaline phosphatase
HIFs: Hypoxia-inducible factor
MSCs: Mesenchymal stem cells
ESC: Embryonic stem cell
FBS: Fetal bovine serum
bFGF: Basic fibroblast growth factor
CCK-8: Cell counting kit-8
EMT: Epithelial-mesenchymal transition

## References

[1] Nguyen PK, Rhee JW, Wu JC (2016). Adult stem cell therapy and heart failure, 2000 to 2016: a systematic review. Jama Cardiol.

[2] Trounson A, Mcdonald C (2015). Stem cell therapies in clinical trials: progress and challenges. Cell Stem Cell.

[3] Kuntin D, Genever P (2021). Mesenchymal stem cells from biology to therapy. Emerg Topic Life Sci.

[4] Wilson AJ, Rand E, Webster AJ, Genever PG (2021). Characterisation of mesenchymal stromal cells in clinical trial reports: analysis of published descriptors. Stem Cell Res Ther.

[5] Guadix JA, Zugaza JL, Gálvez-Martín P (2017). Characteristics, applications and prospects of mesenchymal stem cells in cell therapy. Med Clin.

[6] Hu Y, Lou B, Wu X, Wu R, Wang H, Gao L (2018). Comparative study on in vitro culture of mouse bone marrow mesenchymal stem cells. Stem Cells Int.

[7] Ylostalo JH (2020). 3D stem cell culture. Cells.

[8] Reynolds BA, Weiss S (1992). Generation of neurons and astrocytes from isolated cells of the adult mammalian central nervous system. Science.

[9] Shen FH, Werner BC, Liang H, Shang H, Yang N, Li X (2013). Implications of adipose-derived stromal cells in a 3D culture system for osteogenic differentiation: an invitro and in vivo investigation. Spine J.

[10] Haycock JW (2011). 3D cell culture: a review of current approaches and techniques. Methods Mol Biol.

[11] Li X, Li N, Chen K, Nagasewa S, Yoshizawa M, Kagami H (2018). Around 90° contact angle of dish surface is a key factor in achieving spontaneous spheroid formation. Tissue Eng Part C Method.

[12] Paşca AM, Sloan SA, Clarke LE, Tian Y, Makinson CD, Huber N (2015). Functional cortical neurons and astrocytes from human pluripotent stem cells in 3D culture. Nat Methods.

[13] Gurumurthy B, Bierdeman PC, Janorkar AV (2017). Spheroid model for functional osteogenic evaluation of human adipose derived stem cells. J Biomed Mater Res A.

[14] Li N, Li X, Chen K, Dong H, Kagami H (2019). Characterization of spontaneous spheroids from oral mucosa-derived cells and their direct comparison with spheroids from skin-derived cells. Stem Cell Res Ther.

[15] Chen K, Li X, Dong H, Zhang Y, Yoshizawa M, Kagami H (2019). Spontaneously formed spheroids from mouse compact bone-derived cells retain highly potent stem cells with enhanced differentiation capability. Stem Cell Int.

[16] Sodek J, Mckee MD (2010). Molecular and cellular biology of alveolar bone. Periodontol.

[17] Balic A, Thesleff I (2015). Tissue interactions regulating tooth development and renewal. Curr Top Dev Biol.

[18] Chen C, Tarlé S, Kaigler D (2020). Characterization of the immunomodulatory properties of alveolar bone-derived mesenchymal stem cells. Stem Cell Res Ther.

[19] Akintoye SO (2018). The distinctive jaw and alveolar bone regeneration. Oral Dis.

[20] Liu Y, Wang H, Dou H, Tian B, Li L, Jin L (2020). Bone regeneration capacities of alveolar bone mesenchymal stem cells sheet in rabbit calvarial bone defect. J Tissue Eng.

[21] LingE L, Zhang R, Li C, Zhang S, Ma X, Xiao R (2021). Effects of rhBMP-2 on bone formation capacity of rat dental stem/progenitor cells from dental follicle and alveolar bone marrow. Stem Cells Dev.

[22] Han J, Okada H, Takai H, Nakayama Y, Maeda T, Ogata Y (2009). Collection and culture of alveolar bone marrow multipotent mesenchymal stromal cells from older individuals. J Cell Biochem.

[23] Millman JR, Tan JH, Colton CK (2009). The effects of low oxygen on self-renewal and differentiation of embryonic stem cells. Curr Opin Organ Transplant.

[24] Yeo CD, Kang N, Choi SY, Kim BN, Park CK, Kim JW (2017). The role of hypoxia on the acquisition of epithelial-mesenchymal transition and cancer stemness: a possible link to epigenetic regulation. Korean J Intern Med.

[25] Bhagat M, Palanichamy JK, Ramalingam P, Mudassir M, Irshad K, Choosdol K (2016). HIF-2α mediates a marked increase in migration and stemness characteristics in a subset of glioma cells under hypoxia by activating an Oct-4/Sox-2-mena (INV) axis. Int J Biochem Cell Biol.

[26] Zhang S, Zhao L, Wang J, Chen N, Yan J, Pan X (2017). HIF-2α and Oct4 have synergistic effects on survival and myocardial repair of very small embryonic-like mesenchymal stem cells in infarcted hearts. Cell Death Dis.

[27] Lin W, Li Q, Zhang D, Zhang X, Qi X, Wang Q (2021). Mapping the immune microenvironment for mandibular alveolar bone homeostasis at single-cell resolution. Bone Res.

[28] Zhang Y, Xu Y, Zhou K, Kao G, Yan M, Xiao Y (2021). Hypoxia-inducible transcription factor-1α inhibition by topotecan protects against lipopolysaccharide-induced inflammation and apoptosis of cardiomyocytes. Biomed Eng Online.

[29] Xu J, Zheng L, Chen J, Sun Y, Lin H, Jin R (2017). Increasing AR by HIF-2α inhibitor (PT-2385) overcomes the side-effects of sorafenib by suppressing hepatocellular carcinoma invasion via alteration of pSTAT3, pAKT and pERK signals. Cell Death dis.

[30] Aghali A (2021). Craniofacial bone tissue engineering: current approaches and potential therapy. Cells.

[31] Lv FJ, Tuan RS, Cheung K, Leung V (2014). Concise review: the surface markers and identity of human mesenchymal stem cells. Stem Cells.

[32] Morcos MNF, Schoedel KB, Hoppe A, Behrendt R, Basak O, Clevers HC (2017). Sca-1 expression level identifies quiescent hematopoietic stem and progenitor cells. Stem Cell Rep.

[33] Pealosa-Ruiz G, Mulder KW, Veenstra G (2020). The corepressor NCOR1 and OCT4 facilitate early reprogramming by suppressing fibroblast gene expression. PeerJ.

[34] Karagiannis P, Takahashi K, Saito M, Yoshida Y, Okita K, Watanabe A (2019). Induced pluripotent stem cells and their use in human models of disease and development. Physiol Rev.

[35] Solis-Castro OO, Boissonade FM, Rivolta MN (2020). Establishment and neural differentiation of neural crest-derived stem cells from human dental pulp in serum-free conditions. Stem Cells Transl Med.

[36] Santos JM, Camões SP, Filipe E, Cipriano M, Barcia RN, Filipe M (2015). Three-dimensional spheroid cell culture of umbilical cord tissue-derived mesenchymal stromal cells leads to enhanced paracrine induction of wound healing. Stem Cell Res Ther.

[37] Frith JE, Thomson B, Genever PG (2010). Dynamic three-dimensional culture methods enhance mesenchymal stem cell properties and increase therapeutic potential. Tissue Eng Part C Method.

[38] Jaukovi A, Abadjieva D, Trivanovi D, Stoyanova E, Kostadinova M, Pashova S (2020). Specificity of 3D MSC spheroids microenvironment: impact on MSC behavior and properties. Stem Cell Rev Rep.

[39] Guo L, Zhou Y, Wang S, Wu Y (2014). Epigenetic changes of mesenchymal stem cells in three-dimensional (3D) spheroids. J Cell Mol Med.

[40] Jeon S, Lee HS, Lee GY, Park G, Kin T, Shin J (2017). Shift of EMT gradient in 3D spheroid MSCs for activation of mesenchymal niche function. Sci Rep.

[41] Li S, Ding C, Guo Y, Zhang Y, Wang H, Sun X (2021). Mechanotransduction regulates reprogramming enhancement in adherent 3D keratocyte cultures. Front Bioeng Biotechnol.

[42] Cheema U, Brown RA, Alp B, MacRobert AJ (2008). Spatially defined oxygen gradients and vascular endothelial growth factor expression in an engineered 3D cell model. Cell Mol Life Sci.

[43] Dou L, Yan Q, Liang P, Zhou P, Zhang Y, Ji P (2018). iTRAQ-based proteomic analysis exploring the influence of hypoxia on the proteome of dental pulp stem cells under 3D culture. Proteomics.

[44] Chen G, Liu B, Yin S, Li S, Guo Y, Wang M (2020). Hypoxia induces an endometrial cancer stem-like cell phenotype via HIF-dependent demethylation of SOX2 mRNA. Oncogenesis.

[45] Yeo CD, Kang N, Choi SY, Kim BN, Park CK, Kin JW (2017). The role of hypoxia on the acquisition of epithelial-mesenchymal transition and cancer stemness: a possible link to epigenetic regulation. Korean J Intern Med.

[46] Bhang SH, Cho SW, La WG, Lee TJ, Yang KS, Sun AY (2011). Angiogenesis in ischemic tissue produced by spheroid grafting of human adipose-derived stromal cells. Biomaterials.

