# Supplementary figures and images for: Spontaneous spheroids from alveolar bone-derived mesenchymal stromal cells maintain pluripotency of stem cells by regulating hypoxia-inducible factors
Source: Biol Res. 2023 Apr 5;56:17. doi: 10.1186/s40659-023-00421-w (PMC10074860; doi:10.1186/s40659-023-00421-w)

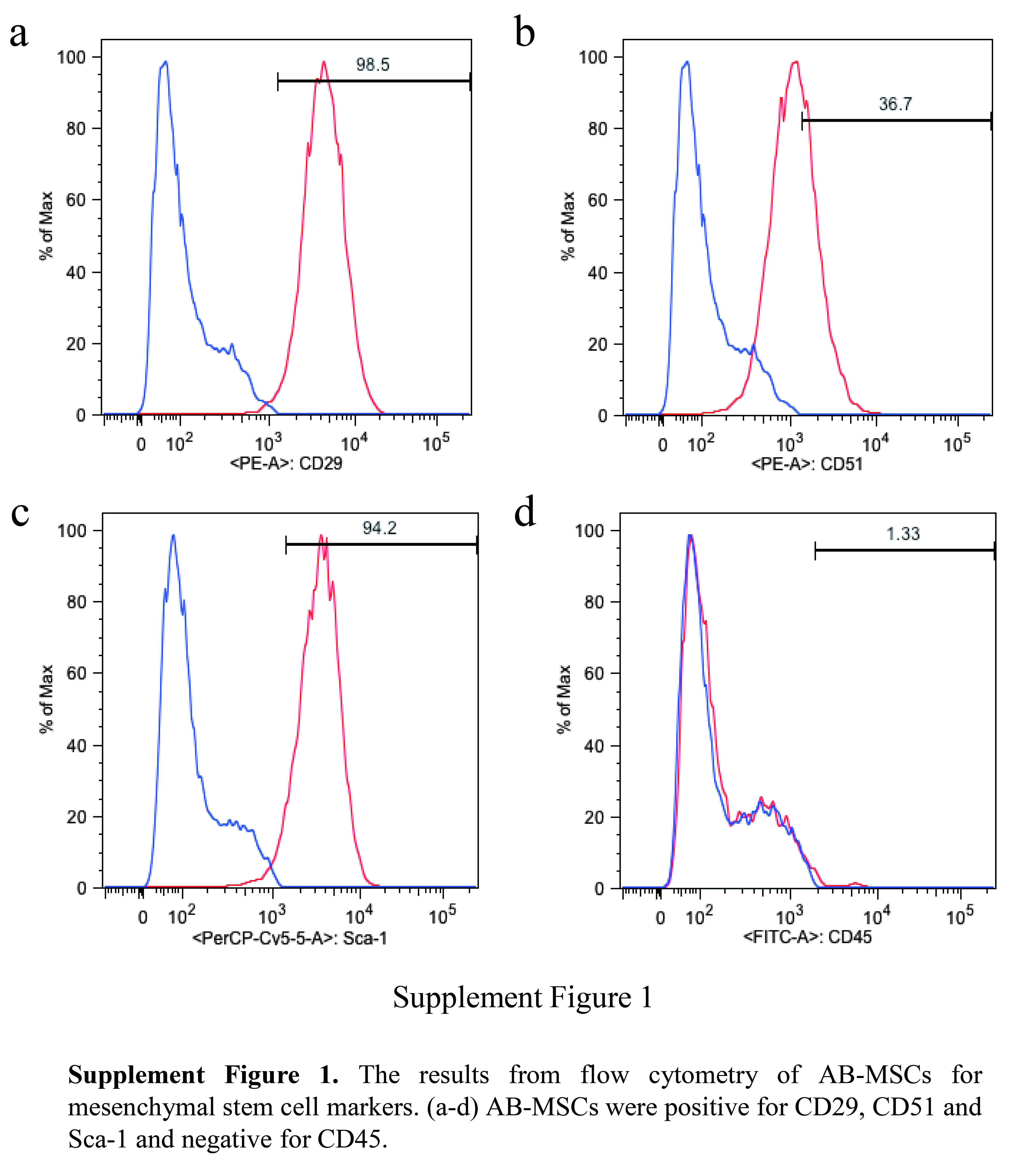

Supplement: Supplementary file 1 — Additional file 1: Figure S1. The results from flow cytometry of AB-MSCs for mesenchymal stem cell markers. (a-d) AB-MSCs were positive for CD29, CD51 and Sca-1 and negative for CD45. [file 40659_2023_421_MOESM1_ESM.tif]
